# First steps to define murine amniotic fluid stem cell microenvironment

**DOI:** 10.1038/srep37080

**Published:** 2016-11-15

**Authors:** E. Bertin, M. Piccoli, C. Franzin, G. Spiro, S. Donà, A. Dedja, F. Schiavi, E. Taschin, P. Bonaldo, P. Braghetta, P. De Coppi, M. Pozzobon

**Affiliations:** 1 Stem Cells and Regenerative Medicine Lab, Fondazione Istituto di Ricerca Pediatrica Città della Speranza, Padova, Italy; 2 Department of Molecular Medicine, University of Padova, Padova, Italy; 3 Department of Cardiac, Thoracic and Vascular Sciences, University of Padova, Padova, Italy; 4 Familial Cancer Clinic and Oncoendocrinology, Veneto Institute of Oncology, Padova, Italy; 5 Stem Cells and Regenerative Medicine Section, Developmental Biology and Cancer Programme, UCL Institute of Child Health and Great Ormond Street Hospital, London, United Kingdom

## Abstract

Stem cell niche refers to the microenvironment where stem cells reside in living organisms. Several elements define the niche and regulate stem cell characteristics, such as stromal support cells, gap junctions, soluble factors, extracellular matrix proteins, blood vessels and neural inputs. In the last years, different studies demonstrated the presence of cKit^+^ cells in human and murine amniotic fluid, which have been defined as amniotic fluid stem (AFS) cells. Firstly, we characterized the murine cKit^+^ cells present both in the amniotic fluid and in the amnion. Secondly, to analyze the AFS cell microenvironment, we injected murine YFP^+^ embryonic stem cells (ESC) into the amniotic fluid of E13.5 wild type embryos. Four days after transplantation we found that YFP^+^ sorted cells maintained the expression of pluripotency markers and that ESC adherent to the amnion were more similar to original ESC in respect to those isolated from the amniotic fluid. Moreover, cytokines evaluation and oxygen concentration analysis revealed in this microenvironment the presence of factors that are considered key regulators in stem cell niches. This is the first indication that AFS cells reside in a microenvironment that possess specific characteristics able to maintain stemness of resident and exogenous stem cells.

Stem cells (SC) are present in all organisms and possess the ability of keeping the undifferentiated state along the life span of a living subject or undergoing differentiation on more specialized cell types after specific stimuli. Fetal SC are generally defined as broadly multipotent SC because they are more prone to turn into different cell types than their adult counterpart. Among those, cKit (CD117) positive cells selected from the amniotic fluid (AF) and defined as amniotic fluid stem (AFS) cells may be relevant to therapeutic approaches because they are easy to access^1^,^2^. AFS cells possess enhanced attitude of growing in culture, along with differentiation ability toward mesoderm, ectoderm and endoderm lineages, and can be reprogrammed without viral transfection^3^. The self-renewal capacity and broad multipotency of AFS cells have been proved not only *in vitro*, but also *in vivo*; in particular it has been demonstrated the ability of AFS cells to replenish the hematopoietic system and the muscle SC niche also after secondary transplantation^4^,^5^, and to participate in mammary gland regeneration^6^ highlighting in the AF the presence of cells with stemness characteristics. According to the studies that have located and characterized the SC niches in mammalian tissues using *in situ* labeling systems, concrete evidences proved that niches are fundamental to maintain SC pool and functions^7^,^8^,^9^. Along the years it has been proved that distinct classes of niches harbor different SC such as the hematopoietic, the neural and the mesenchymal (epidermis, gut and skeletal muscle)^8^,^9^,^10^,^11^,^12^. Postnatal hematopoiesis occurs mainly in the bone marrow in the best characterized SC niche, where hematopoietic stem and progenitor cells reside^13^. The niche is responsible to define the microenvironment where quiescent SC are located before specific signals accomplish the dormant state and activate differentiation process. In particular, this dynamic compartment fulfils mainly three functions through secreted or cell surface molecules: it controls SC proliferation, determines the fate of SC daughters and protects SC from exhaustion or death.

It is known that there are common elements constituting the niche: stromal cells that support SC interacting with each other via cell surface receptors and soluble factors, extracellular matrix (ECM) proteins that supply structural organization and mechanical signals to the niche, vasculature and nervous system that drag systemic and physiological inputs^14^. The role of ECM is of paramount importance because, on one hand, the interactions with ECM provide essential mechanical cues^14^ and, on the other, the ECM can concentrate growth factors and cytokines by binding both local and systemic biomolecules within the niche^15^. Among all the physiological stimuli, oxygen tension has emerged as an important component in different niches, indeed SC that reside in a hypoxic niche possess slow-cycling proliferation rates while avoiding the oxidative stress^16^,^17^,^18^,^19^.

The SC in the niche are quiescent and at the same time able to promptly become active because of a global balance among all components and signals; consequently, deregulation of this complex equilibrium causes niche dysfunction with development of diseases associated with aging^20^,^21^, tumorigenesis^22^,^23^ and tissue degeneration^24^. Hence, cells within the niche represent possible pharmacological targets with therapeutic potential for some diseases.

In the present work, as first study that aim to investigate the characteristics of the extraembryonic microenvironment, we analyzed the cKit positive cells isolated from the AF and amnion (AM) of mouse embryos. To start, we established an *in vivo* model of in utero transplantation (IUT) with mouse YFP^+^ embryonic stem cells (ESC) used as tool to analyze whether AF and AM were able both to maintain the characteristics of YFP^+^ ESC and to be a specific environment for extraembryonic SC reservoir.

## Results

### Characterization of extraembryonic mouse cKit^+^ cells

**Figure 1:**
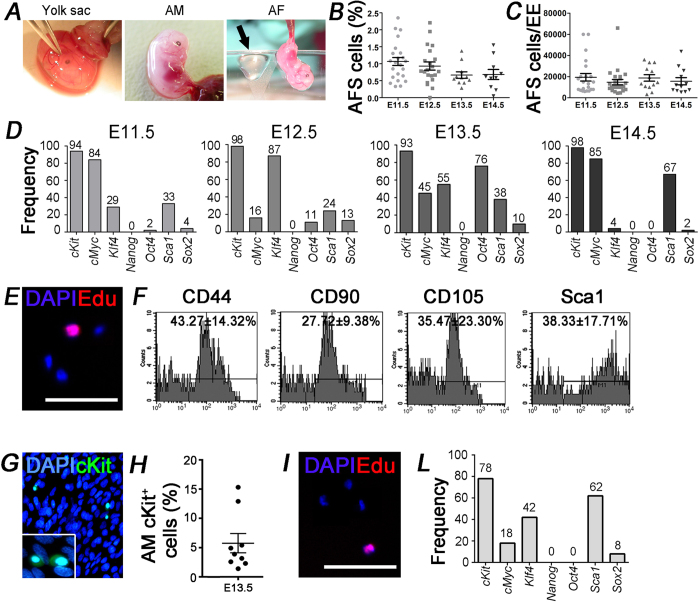
Characterization of cKit^+^ cells isolated from the murine AF and AM. (**A**) Amniocentesis procedure, from the removal of the fetal membrane to the collection of the AF. (**B**) The percentage of AFS (cKit^+^) cells at different embryonic stages. Mean is represented by bar. (**C**) Total number of AFS cells per embryo equivalent (EE). Mean is represented by bar. (**D**) Frequency of AFS cells positive for the expression of each gene (*cKit*, *cMyc*, *Klf4*, *Nanog*, *Oct4*, *Sca1*, *Sox2*) at different embryonic stages evaluated by single cell PCR (E11.5: 49 cells (n = 7 embryos); E12.5: 38 cells (n = 9 embryos); E13.5: 42 cells (n = 31 embryos); E14.5: 52 cells (n = 10 embryos)). (**E**) Representative immunostaining showing some EdU^+^ cells among the cKit^+^ cells isolated from AF (n = 7 embryos). (**F**) Flow cytometry analysis highlighted the presence of AFS cells positive for the markers CD44, CD90, CD105 and Sca1 (n = 31 embryos). (**G**) Representative immunostaining showing cKit^+^ cells in the AM (n = 3 embryos). (**H**) Percentage of cKit^+^ cells isolated from AM. (**I**) EdU^+^ cells among the cKit^+^ cells isolated from AM (n = 7 embryos). Scale bars = 100 μm. (**L**) Frequency of E13.5 ckit^+^ AM cells positive for the expression of each gene (*cKit*, *cMyc*, *Klf4*, *Nanog*, *Oct4*, *Sca1*, *Sox2*) studied by single cell PCR (50 cells (n = 18 embryos)).

At first, using the amniocentesis procedure (Fig. 1A) we isolated cKit^+^ cells from AF at different embryonic stages in a time window corresponding approximately to the second trimester of human gestation. In all the analyzed samples, AFS cells were present in a variable proportion according to the gestational age (Fig. 1B,C). Specifically at E11.5 in AF there was a peak in the percentage of AFS cells that slowly decreased in the next days (Fig. 1B) without significant differences. However, the total number of AFS cells per embryo equivalent (10.000–20.000 cells per embryo, Fig. 1C) was similar in all the analyzed time points, suggesting the idea that AFS cells are present in equal number in each AF until birth. After isolation, we investigated the frequency of SC marker expression at single cell level by multiplex PCR, from E11.5 to E14.5 (Fig. 1D), showing that *cMyc*, *Klf4*, *Sca1* and *Sox2* were expressed in all the analyzed embryonic stages, even if at different frequency. Interestingly *Oct4* was present in 2%, 11% and 76% of cells respectively at E11.5, E12.5 and E13.5, while there were no positive cells at E14.5. Cells isolated at E13.5 displayed the expression of pluripotency genes *Oct4*, *Sox2*, *cMyc* and *Klf4* on a considerable frequency (76%, 10%, 45% and 55%, respectively) and the majority of the cells co-expressed more than one gene (Table 1). For these reasons, we focused all the further experiments at this gestational age. We discovered only few proliferating EdU^+^ cells (4.5 ± 10%, Fig. 1E) and according to the immunophenotype, the majority of cKit^+^ cells co-expressed mesenchymal markers such as CD44, CD90, CD105 and Sca1 (Fig. 1F, supplementary Fig. 1). Since the contiguity between AF and AM, we decided to investigate whether it was possible to isolate cKit^+^ cells also from this membrane^4^. This population represented 5.8 ± 4.9% of total cells at E13.5 (Fig. 1G,H) and about 17 ± 36% of AM cKit^+^ cells were EdU^+^ (Fig. 1I). We then analyzed the frequency of SC marker expression at single cell level and detected a lower percentage of cells positive for *cMyc* (18%) and *Klf4* (42%) (Fig. 1L) in respect to cells in the AF at the same gestational age (Fig. 1D), but most importantly no *Oct4* positive cells were found in cKit^+^ AM population (Fig. 1L), indicating a more committed phenotype of cKit^+^ cells isolated from the membrane in respect to those floating in the fluid.

### Characterization of extraembryonic mouse environment

**Figure 2:**
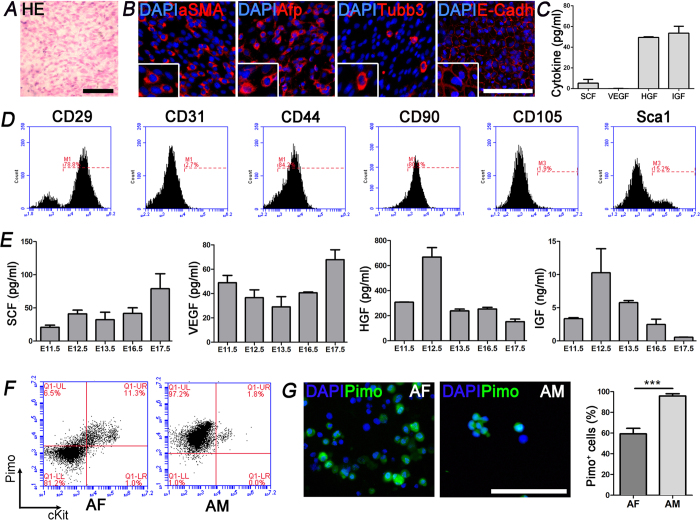
The extraembryonic stem cells niche: AM, cytokines and hypoxia. (**A**) Histological analysis of the AM and (**B**) representative images of immunostaining for αSMA, Afp, Tubb3 and E-Cadherin (n = 5 embryos). (**C**) Cytokines detection and analysis on AM obtained at the embryonic day 13.5. Analyzed cytokines: mouse SCF; mouse VEGF; mouse HGF; mouse IGF (n = 25 embryos). (**D**) AM flow cytometry analysis highlighted the presence of cells positive for the mesenchymal markers CD29, CD44, CD90 and Sca1 and negative for CD31 and CD105 (n = 20 embryos). (**E**) Cytokines detection and analysis on AF obtained at different embryonic days (E11.5, E12.5, E13.5, E16.5 and E17.5). Analyzed cytokines: mouse SCF; mouse VEGF; mouse HGF; mouse IGF (n = 60 embryos). (**F**) Flow cytometry analysis confirmed the presence of Pimo^+^ cells in the AF and AM (n = 6 embryos). (**G**) Immunostaining of Pimo demonstrated the presence of different percentage of hypoxic cells between AF and AM. (***p < 0.01) (n = 8 embryos). Scale bars = 100 μm.

In order to better characterize the SC population residing in AF, we sought to deeper analyze the environment in which these cells live for a physiologically time-restricted period. Mouse AM is an avascular membrane composed of two cell monolayers that express developmental markers, such as αSMA, Afp, Tubb3, the adhesion molecule E-Cadherin (Fig. 2A,B), and typical markers of stromal cells like CD29, CD44, CD90 and Sca1 (Fig. 2D, supplementary Fig. 1). We did not detect expression of specific endothelial markers such as CD31, neither the presence of the angiogenic cytokine VEGF (Fig. 2C). Differently, the AM seems to be rich in other molecules, which have typically an effect on cell growth and migration such as SCF, HGF and IGF (Fig. 2C). Interestingly, the same cytokines were monitored also in the AF during the course of the murine gestation, from E11.5 to E17.5, and were all present with a specific trend, suggesting a very complex and orchestrated signal environment (Fig. 2E). Since oxygen tension plays a crucial role in SC homeostasis especially in the niche, we investigated this aspect regarding AM and AF cells. To this aim, the hypoxic marker piminidazole (Pimo) was injected in tail vein of pregnant females, which were sacrificed 2 hours post injection. When Pimo is administered *in vivo*, it forms stable adducts in hypoxic regions that can be subsequently identified with an anti-Pimo antibody. We evaluated the presence of Pimo positive cells by flow cytometry and immunofluorescence, both in AF and AM, and bone marrow was used as positive control (data not shown). Flow cytometry revealed 11.3% and 1.8% of Pimo^+^ ckit^+^ cells respectively in the AF and in the AM (Fig. 2F). Regarding the cKit positive population we found 88.9% of Pimo^+^ cells in the AF and 91.9% in the AM. When we evaluated the hypoxic profile by immunofluorescence, we detected 95.9 ± 2.1% of Pimo^+^ cells in the AM (Fig. 2G), similar to the value observed by flow cytometry, and 59.3 ± 5.3% of Pimo^+^ cells in the AF (Fig. 2G), slightly different to the analysis using flow cytometry but still pointing out that the majority of the AFS cells are in a hypoxic condition.

### IUT injection of ESC

**Figure 3:**
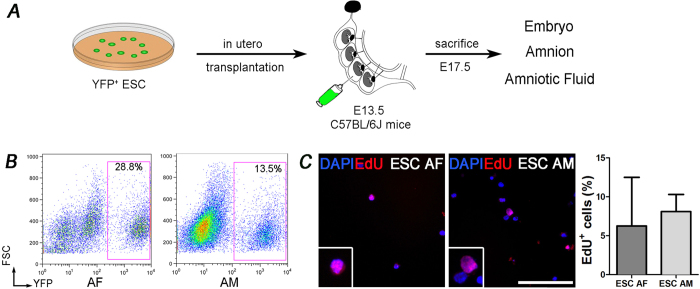
Experimental plan and *in vivo* cell detection. (**A**) YFP^+^ ESC were injected into the amniotic cavity of embryos at E13.5 and 4 days after injection AF, AM and embryos were analyzed (n experiments = 24). (**B**) FACS analysis of cells isolated from AF and AM after IUT (n = 16 embryos). (**C**) Immunostaining and quantification of EdU^+^ cells among all the YFP^+^ ESC isolated from AF and AM after IUT (n = 8 embryos).

We performed IUT experiments (listed in Table S1) injecting 10^5^ YFP^+^ ESC into the amniotic cavity of E13.5 wild type embryos and then analyzed the YFP^+^ cells isolated from AF, AM and embryos 4 days after injection (i.e. E17.5, Fig. 3A). To design the injection strategy we performed IUT experiments with different number of ESC injection: 10^4^, 10^5^ and 10^6^ (experiments n = 6) per embryo, and, while 10^4^ cells were not sufficient to perform analyses after transplantation, in utero injection of 10^6^ cells caused death of embryos (21% overall survival compared to 82% survival with injection of 10^5^ ESC, Table S1). Then we set 10^5^ cells as standard cell number to be injected in the AF at E13.5. To be able to retrieve AF and to avoid delivery that happen at E19.5, 4 days after IUT AF and AM were collected: we detected YFP^+^ cells ranging from 0.3 to 28.8% in the AF and from 1.2 to 13.5% in the AM (Fig. 3B). YFP^+^ ESC were also analyzed under the proliferative and apoptosis aspect: we identified around 6.3 ± 6.2% and 8.1 ± 2.2% EdU^+^ cells in the AF and AM respectively (Fig. 3C). These data suggest that ESC reside in the AF and AM mainly in a non-proliferative or quiescent state, with a small number of cells in active proliferation. Despite Tunel assay did not evidenced apoptotic or dead YFP^+^ cells (data not shown), we were able to recover about 10^4^ ESC from each embryo (1000–2000 ESC from AF and 8000–9000 ESC from AM), 10 times less than the injected cells. Nevertheless, this cell number was sufficient to perform all the analyses. These numbers are referred to YFP positive cells, although randomly ESC loose, for some unknown mechanism, the YFP expression when *in vitro* expanded (data not shown) and as consequence it can not be excluded to underestimate the number of ESC rescued after IUT. We decided to use ESC not for therapeutic purposes but only as experimental tool being the best-defined SC source, nevertheless, since fetus is part of the microenvironment, we asked whether injected YFP^+^ ESC were located only within the AF and AM or integrated in the embryo tissues eliciting modifications in the mouse development. At first, histological analysis highlighted that the embryo structure was not impaired by cell injection since no organ abnormalities were detected (supplementary Fig. 2A) even in tissues analyzed six weeks after birth (supplementary Fig. 2C). Secondly, we evaluated YFP presence by PCR and by immunofluorescence analysis and in 3 out of 13 analyzed embryos YFP expression was detected (supplementary Fig. 2A,B), whereas rare ESC were found only within the airway spaces (supplementary Fig. 2A).

### ESC maintain pluripotent characteristics after IUT

**Figure 4:**
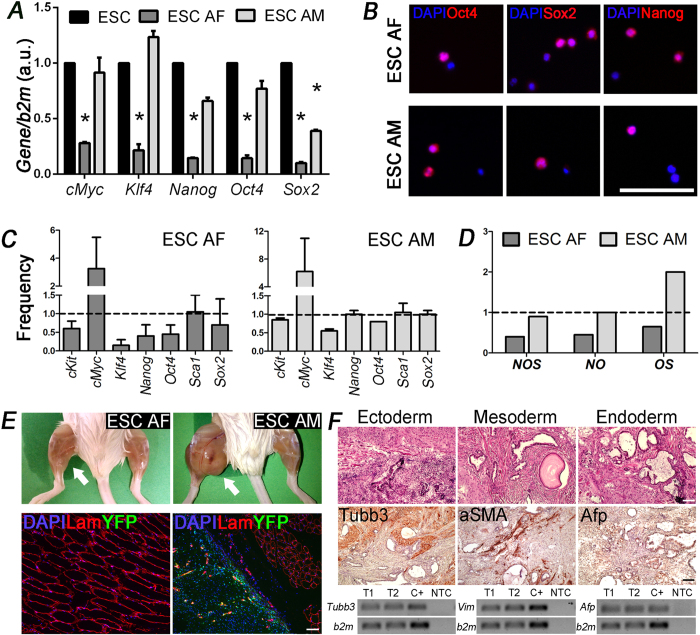
Characterization of YFP^+^ ESC after IUT and *in vivo* teratoma formation. (**A**) PCR analysis of pluripotency markers (*cMyc*, *Klf4*, *Nanog*, *Oct4*, *Sox2*) on YFP^+^ ESC populations before IUT (black bars) and after IUT obtained from AF (dark grey) and AM (light grey) (n = 34 embryos). (*p < 0.05) (**B**) Characterization of stem cell protein expression in ESC after retrieval and FACS sorting from AF and AM (n = 26 embryos). (**C**) Frequency of cells positive for the expression of each gene in the YFP^+^ ESC after IUT obtained from AF (dark grey; 32 and 50 cells (n = 25 embryos) and AM (light grey; 18 and 44 cells (n = 31 embryos), considering YFP^+^ ESC before IUT (53 and 51 cells) as 1. (**D**) Percentage of cells co-expressing each gene (N = *Nanog*; O = *Oct4*; S = *Sox2*) in the analyzed populations. (**E**) Macroscopic aspect of Rag2^−/−^γc^−/−^ mice hindlimbs sacrificed 6 weeks after injection of YFP^+^ ESC post IUT (n = 100 emrbyos). Tumor masses were visible only in mice transplanted with YFP^+^ ESC obtained from AM; immunostaining of YFP and Laminin confirmed the absence of ESC cells in muscle transplanted with YFP^+^ cells isolated from AF, while tumor masses obtained from ESC AM resulted positive for YFP expression. (**F**) Histology and immunostaining of teratomas confirmed the cell differentiation into all three germ layers (ectoderm, mesoderm and endoderm). PCR analysis of two teratoma samples (T1 and T2) obtained after injection of ESC AM. Tumor masses resulted positive for the expression of *β3 tubulin (Tubb3*, ectoderm), *vimentin (Vim*, mesoderm) and *αfetoprotein (Afp*, endoderm). C+ = positive control, NTC = not template control. Scale bars = 100 μm.

Since ESC may spontaneously differentiate in absence of proper stimuli (supplementary Fig. 3), it was of paramount importance to establish if ESC maintained pluripotent state after IUT. Therefore in YFP^+^ ESC isolated from AF (ESC AF) and AM (ESC AM) PCR and immunofluorescence analyses were performed. As shown in Fig. 4A, the gene expression levels of *cMyc*, *Klf4*, *Nanog*, *Oct4* and *Sox2* detected in ESC AF decreased significantly compared to ESC before injection. On the contrary, the expression profile of ESC AM was similar to that of the ESC before IUT (Fig. 4A), suggesting a possible role of the AM for pluripotency maintenance. We also analyzed at single cell level the expression of each stemness gene in ESC AF and AM 4 days after injection. While cells isolated from AM proved to be similar to the control ESC in terms of frequency of SC markers expression, ESC AF reduced the expression frequency of all the studied genes (Fig. 4C). Furthermore, the percentage of cells co-expressing two or more pluripotent genes was similar between ESC before IUT and those isolated from AM, whereas only in few ESC AF *Oct4* and *Nanog* were simultaneously expressed (Fig. 4D). Interestingly, after isolation, both ESC AF and AM retained the expression at protein level of the pluripotency markers Oct4, Sox2 and Nanog (Fig. 4B). To investigate whether ESC AM and AF diverged also in the ability to differentiate into cells of the three germ layers as well as in terms of gene expression both at population and single cell level, we injected IUT YFP^+^ ESC in the hindlimbs of immunocompromised mice. In more detail, ESC post-IUT were collected keeping distinguished those isolated from AF and AM, and injected into the inner part of leg of Rag2^−/−^γc^−/−^ mice. We already tested the inability of AF and AM cells to form teratoma (data not shown) and for this reason, ESC after IUT were not FACS sorted since any tumor mass that would appear would have been the consequence of the injected pluripotent ESC. Moreover ESC after IUT were not *in vitro* expanded before the teratoma assay, but directly injected in the muscle immediately after collection. The injected ESC, obtained after IUT, were not less than 10^5^ since we isolated them from 100 transplanted embryos. To be sure about the minimum ESC (pre-IUT) number sufficient to generate a teratoma, we injected mice with 10^4^, 10^5^ and 10^6^ ESC pre-IUT and identified the presence of tumor masses 6 weeks after injection only in the condition with 10^5^ and 10^6^ cells (supplementary Fig. 3B). We identified that ESC AF were not able to generate tumor, instead ESC AM gave rise to defined masses as highlighted in Fig. 4E by the gross appearance and the YFP immunostaining (Fig. 4E). The tumors were composed of cells displaying expression of genes characteristic of all three germ layers, as evidenced by histological and PCR analyses (Fig. 4F). Taken together, these results clearly indicate that only ESC attached to the AM after the 4 days in the amniotic cavity were able to maintain their pluripotency in terms of SC gene expression and ability to form differentiated cells from mesoderm, ectoderm and endoderm embryonic layers. Due to these results we hypothesized that AM acts as stromal support for the stemness maintenance of ESC, as showed also in other studies which used AM as *in vitro* stromal support^25^,^26^. We performed the staining of ESC expanded *in vitro* for 4 days (same time interval as for IUT transplantation) onto the whole AM, obtained from embryos at E13.5, in a basal medium (without LIF). ESC in this condition formed the characteristic colonies, which were positive for the expression of Oct4, Sox2 and Nanog (supplementary Fig. 4). These findings validate our hypothesis that considers AM as stromal support.

### IUT injection of AFS cells

**Figure 5:**
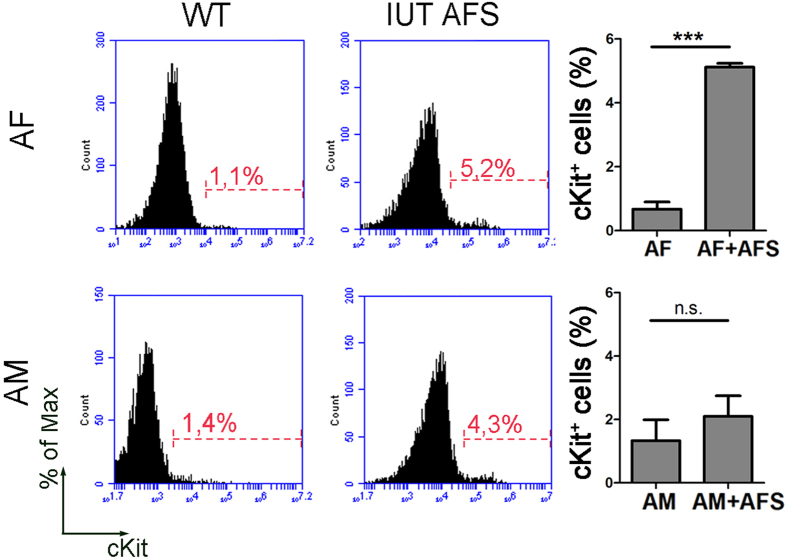
Detection of AFS cells after IUT. Flow cytometry analysis and quantification of cKit^+^ cells isolated from AF and AM at E17.5 in the non-injected (WT) and injected (IUT AFS) condition. (***p < 0.01) (n = 7 embryos).

On the basis of the results obtained with the gene expression analysis of the cKit^+^ cells from AF and AM (Fig. 1), which showed that AFS cells had a higher frequency of pluripotency marker expression in respect to homologous cells isolated from the AM, we decided to perform AFS IUT. We transplanted 10^4^ AFS cells per embryo (n = 7 embryos), following the same procedure used for ESC. We evaluated, by flow cytometry analysis, the percentage of cKit^+^ cells identified at E17.5 in the AF and AM, comparing between non-injected (defined WT) and injected (IUT AFS) (Fig. 5). We pointed out an increase of cKit^+^ cells in the AF after IUT, suggesting that AFS seem to prefer the AF localization in respect to the AM, on which only a low percentage of cells adhered during the experimental period.

## Discussion

CD117/cKit is a marker that identifies the SC population in the AF, namely the AFS cells; we investigated the microenvironment where these cells are floating to seek whether the fundamental elements forming a SC niche could be uncovered. At first, we performed gene expression analysis in cKit positive cells isolated from AF, showing that the embryonic stage 13.5 was the best time point in terms of pluripotency markers expression. Single cell PCR analysis, previously used to identify variable gene expression of ESC colonies^27^, revealed the genetic heterogeneity of AFS cell population.

Then we compared cells from AF and AM, at E13.5, seeing that AFS cells had a higher frequency of pluripotency markers expression in respect to homologous cells isolated from the AM. This finding indicated a higher stemness level of cKit^+^ cells in AF in comparison to the population identified in the surrounding membrane. We previously demonstrated the ability of mouse AFS cells to differentiate into different tissues (muscle and hematopoietic)^4^,^5^ but they are not pluripotent cells. In fact, when injected into an immunocompromised mouse they do not form teratoma. An analogous result was obtained with cells isolated from AM. Since Nanog together with Yamanaka factors, plays a pivotal role in the establishment of the pluripotent state and, in concert with Oct4, also in its maintenance^28^,^29^,^30^, the absence of its expression leads us to define these cKit positive cells as broadly multipotent rather than pluripotent.

Given that AFS cells own SC behavior and express stemness markers, we wondered if these characteristics are maintained *in vivo* by the extraembryonic microenvironment. Therefore we studied the AM, observing that this membrane conveys different stromal markers, including E-Cadherin, a fundamental protein for SC anchorage to the niche^31^. Secondly, we evaluated the presence and the concentration of cytokines and growth factors that were demonstrated to play a central role also in other SC niches. We detected SCF, which is a master regulator of the self-renewal and differentiation in the hematopoietic niche^32^, HGF and IGF-1, both key factors for the expansion, self-renewal and differentiation of the defined niche of satellite cells^33^, and VEGF which is involved in cell proliferation during embryogenesis^34^. Oxygen tension evaluation by means of the marker Pimo, which detects cells that experience an oxygen concentration around 1.3% (10 mmHg), revealed that cKit^+^ cells both in the AM and AF live in low oxygen tension (hypoxia) environment. Together with structural and soluble factors, hypoxia is essential for the stemness maintenance of embryonic, hematopoietic, mesenchymal and neural SC, and could also influence proliferation and cell-fate commitment^35^.

We are aware that this is the first study facing the aspect of defining the extraembryonic SC niche, indeed more than fifty years of research investigations have been dedicated to the most studied SC niche such as the hematopoietic one^9^. So far, all the analyzed aspects suggested that the extraembryonic environment displays features that could parallel it to a specific niche. However, to really prove this, it is necessary to deplete the extraembryonic environment of its own SC and demonstrate the ability to accept and maintain a newly introduced SC population^36^. Indeed, this is what happens when hematopoietic SC from a donor replenish the irradiated bone marrow of a host organism or when an empty Drosophila SC niche attracts foreign somatic SC and transiently maintain their characteristics^37^. In this view, although in the future we plan to study the way of AFS cells depletion, we decided to use an exogenous SC population such as mouse ESC^38^,^39^ as a tool to investigate the possible existence of an extraembryonic SC niche by their injection into the amniotic cavity. Importantly, mouse ESC proved to be a good tool since these cells can maintain the pluripotency also when are floating. A protocol published by *Andang et al.*^40^ showed that with the presence of correct stimuli these cells keep their characteristics also when are in suspension. In addition, it has already been demonstrated that fetal microenvironment may participate in the control of ESC growth^41^, indicating that there are strong differences between adult and fetal environment. Usually, when ESC are transferred into a foreign environment, an uncontrolled differentiation program is activated leading to the formation of a disorganized multicellular mass, which contains a multiplicity of cell types, namely teratoma^42^. The absence of an appropriate microenvironment created by specific intercellular interactions, systemic inputs and cellular organization, causes the disorganized ESC differentiation. Conversely, when ESC are injected into their native niche, or rather a recipient blastocyst, they contribute to the generation of all tissues of the new organism^43^. In the last decades, *in vitro* experiments were performed to establish which conditions and inputs are necessary to maintain pluripotency when ESC are cultured in a dish, allowing their expansion and controlled differentiation for a wide range of purposes. An ESC culture bases on a dynamic equilibrium between cells that self-renew and others that differentiate mainly under the control of a small transcription factor network with three core components: Nanog, Oct4 and Sox2^28^. This fragile equilibrium is highlighted by the stemness gene expression at single cell level: not all the cells co-expressed the pluripotency genes^27^,^44^,^45^,^46^, but this is in step with the fact that quiescence, proliferation and differentiation processes are dynamic events inside a SC population. It is important to underline that we used ESC to investigate the capacity of the extraembryonic environment to maintain the characteristics of foreign cells and not for therapeutic purposes; considering this aim, E13.5 was the suitable gestational age to perform IUT because the embryo at this stage has the dimension to allow the injection into the amniotic cavity without damaging the fetal structures. Moreover the majority of the fetal organs are completing their development, giving us an higher probability that cells would stay in the AM and AF without been attracted by the embryo. When we injected YFP^+^ ESC into the amniotic cavity, we observed that they did not integrate into the embryos, probably because at the embryonic stage of 13.5 there are not the mandatory stimuli for ESC to take part to the organogenesis, and we did not detect tumor masses in mice born after IUT. In addition, only a small number of ESC were EdU^+^, to indicate the absence of proliferation inputs, as it occurs in other niches^47^, and we did not found apoptotic cells. For these reasons, we believe that ESC were accepted from the extraembryonic environment, as confirmed also by the high survival rate (82%) of the embryos after IUT. On the contrary, when we injected ESC in the fetal abdominal cavity, a liquid microenvironment structurally similar to the AF but characterized by different stimuli, we did not retrieve any YFP^+^ cells (data not shown). This is in keeping with several published works demonstrating that cells intraperitoneally and intravenously injected at E13.5 are rejected in immunocompetent fetuses^48^,^49^,^50^,^51^. Following these results, we can underline that, in immunocompetent mice (such as our model), there is a strong difference between IUT of ESC injected in the AF or intraperitoneally in the fetus. In the first condition, ESC are rescue while in the fetus cells are rejected due to lack of immunotolerance. Moreover, the fact the ESC are not rejected in the AF indicates that this is also a privileged environment.

When we analyzed ESC isolated after IUT we found that ESC floating in the AF strongly reduced the gene expression of the pluripotency markers and lost the ability to form teratoma, while this did not occur to ESC attached to the AM. These findings suggest a role of the AM as stromal support able to maintain ESC characteristics, as demonstrated when we cultivated ESC onto the whole AM *in vitro*. Amniotic membrane is rich in stromal cells and secretes cytokines and growth factors, such as SCF, HGF and IGF, that are produced by cell lines (i.e. STO and CF-1) usually employed as feeder layer *in vitro* for ESC expansion^52^. Moreover, *Bashamboo et al.*^53^ showed that *in vitro* ESC differentiation is dependent on the SCF-KIT pathway, and *Chen et al.*^54^ demonstrated how a signalling by vitamin A/retinol promotes the self-renew of ESC through the activation of PI3K/Akt signalling via IGF-1 receptor. According to what has been shown for the Drosophila germ stem cell (GSC) niche, the direct interaction between SC and somatic neighbors is fundamental for the stemness maintenance. In the GSC niche, the responsible of the intercellular connection and cross-talk are cadherins and catenins, which constitute the adherent junctions that are the physiological link between cells and stroma. Molecular studies proved that mutations in these junctions lead to the inability of the somatic cells in recruiting and maintaining the GSC^55^. Moreover, E-cadherin seems to play an essential role in balancing ESC self-renew and differentiation^56^, and for this reason the E-Cadherin expression by AM cells could further sustain the hypothesis of a role as stroma support. Following this idea, different studies already showed the ability of AM membrane to sustain stemness characteristics *in vitro*^25^,^26^. A more extensive investigation to identify which signaling pathways could be responsible of the maintenance for pluripotency of ESC in the AM will be necessary to better understand the extraembryonic microenvironment. In the prospective stemness of AFS cells, it could be hypothesized that the AM is more important for the cytokines and soluble factors produced rather than for its stromal support, since the AFS cells are naturally floating in the AF. Indeed when we injected AFS cells, more cells were found on AF rather than AM. The process of floating in AF and attaching to AM of both injected ESC and endogenous AFS cells is probably dynamic. Nevertheless, the two cell types are different: AFS cells are not pluripotent and physiologically floating, ESC are pluripotent and naturally grow in cell agglomerates (blastocyst) or colonies. Indeed, after IUT we found each cell type according to their stemness: broadly multipotent AFS cells in AF and pluripotent ESC on the AM stromal support.

In conclusion, having already demonstrated the ability of AFS cells to both replenish and stimulate a damaged niche^5^,^57^, here we showed the ability of the extraembryonic environment, delimited by the embryo and its membranes, to sustain exogenous SC features. It has been proved^58^ that the characteristics elicited by this environment, such as soluble factors and low oxygen levels (hypoxia), are essential regulators for the maintenance of AFS cell stemness. Further experiments are necessary to establish the physiological role of AFS cells and they interaction with the microenvironment, but starting with these evidences we believed that AM and AF could be considered the murine extraembryonic SC niche.

## Methods

### Mice

All surgical procedures and animal husbandry were carried out in accordance with international guidelines, with the National Institutes of Health Principles of Laboratory Animal Care (National Institutes of Health publication 85–23, revised 1985) and were also approved by the local ethics committee for animal care of the University of Padova (organismo per il benessere degli animali, or OPBA). C57BL/6 J pregnant mice were used for cell isolation and transplantation and Rag2^−/−^γc^−/−^ mice for teratoma assay.

### Amniotic fluid, amnion collection, cell selection and flow cytometry

Embryo age was defined relative to the morning of vaginal plug discovery (E0.5). All dissections were performed under a stereomicroscope (Leica Microsystems) and AF was collected following the procedure described elsewhere^4^.

During the amniocentesis also AM membranes were collected, AM were stretched on a glass slide and fixed for immunostaining or digested with 0.2% collagenase I (Sigma-Aldrich) supplemented with 3% fetal bovine serum (FBS, Life Technologies) to isolate cells. cKit/CD117 positive cells (from AF and AM) were isolated using the Miltenyi Mouse Lineage Cell depletion kit and then CD117 Microbeads (all from Miltenyi Biotech). Cells were counted using a Burker chamber, characterized by the presence of cell markers such as CD44, CD90, CD29, Sca1, CD31, and CD105 (all from BD-Biosciences) and analyzed with Accuri C6 Flow Cytometer (Becton Dickinson). Rat IgG 2a FITC-, rat IgG2a PE- and rat IgG2b APC-Isotype were used as negative control. Total number of AFS cells per embryo equivalent (EE) was defined as the number of cKit^+^ cells divided for the number of embryos.

### Mouse YFP embryonic stem cells culture

Mouse YFP^+^ embryonic stem cells (YFP^+^ ESC), produced by Janet Rossant and Dr. John Roder’s laboratory, were kindly provided by Dr. Andras Nagy and Kat Hadjantonakis of Mount Sinai Hospital in Toronto. YFP^+^ ESC were maintained for different passages under standard culture conditions, on inactivated mouse embryonic fibroblasts (MEF) and in the presence of LIF^59^. Cells were detached and prepared for transplantation. YFP^+^ ESC were also maintained onto whole AM for 4 days, in a basal medium without LIF.

### Assessment of the hypoxic profile

The hypoxic status of AM and AF cells was assessed using Hypoxyprobe-1 Plus Kit (HPI). Pregnant mice (E13.5) were intravenously injected with 120 mg kg^−1^ of piminidazole (Pimo) 2 hours before euthanasia. Cells obtained from AF and AM were used for FACS analysis or stained. For the FACS analysis, cells were permeabilized using IntraPrep Kit (Beckman Coulter) following the manufacturer’s procedure, while for immunofluorescence onto cytospin AF or AM cells were fixed using 4% PFA and permeabilized with 0.1% triton X-100. Detection of intracellular Pimo adducts was performed labeling with FITC-conjugated mouse anti-Pimo antibody. PBS-injected mice were used as controls to detect baseline levels of anti-Pimo antibody binding.

### In utero transplantation and sample preparation

Dams at E13.5 were anesthetized using 3% isofluorane in oxygen. A midline laparotomy was performed to expose uterus and 10 μL of PBS containing 1 × 10^5^ YFP^+^ ESC or 1 × 10^4^ AFS cells were injected within AF of each embryo using hamilton syringe fitted with a 33 G needle. After transplantation analgesic was administered into abdominal cavity before suturing.

After 4 days, at E17.5, every single embryo was isolated and AM and AF were collected. AM membranes were digested with collagenase I as previously described in “Amniotic fluid, amnion collection and cell selection”; AF was centrifuged to separate the cells. AM and AF cells were sorted with FACSAria (Becton Dickinson) for the expression of YFP or cKit (7-Amino-actinomycin D - 7AAD (BD-Biosciences) was added as a viability marker in the sorting procedure) and isolated ESC were analyzed. Embryos were paraffin embedded or snap-frozen for histology, immunostaining and PCR analyses. In one experiment mice were sacrificed after IUT, at six weeks after birth, organs were collected and snap-frozen for histology or immunofluorescence analysis.

### Analysis of EdU incorporation

Pregnant female mice (E13.5 and E17.5 post-IUT) were injected intraperitoneally with 0.5 mg of EdU (Click-iT Edu Imaging Kit, Life Technologies). Cells were collected from AF and AM 3 hours after the injection, in the case of IUT ESC were sorted for YFP, fixed using 4% PFA and then cytospinned. Immunofluorescence has been performed following manufacturer’s instructions.

### Cytokines analysis

Stem cell factor (SCF MCK00, R&D Systems), vascular-endothelial growth factor (VEGF MMV00, R&D Systems), hepatocyte growth factor (HGF MHG00, R&D Systems) and insulin growth factor (IGF-1 MG100, R&D Systems) were analyzed in the AF at different gestational ages and AM at E13.5, using specific ELISA quantikine kit.

### DNA/RNA extraction

DNA from embryos was extracted with a DNeasy Blood & Tissue kit (QIAGEN). RNA from YFP^+^ ESC before and after IUT, teratoma and positive controls (muscle, fetal liver, embryos, brain) was extracted using RNeasy Mini Kit (QIAGEN) following the manufacturer’s instructions.

RNA and DNA were quantified with a Nanodrop ND-2000 spectrophotometer (Thermo Scientific).

### PCR and Realtime PCR

GFP^+^ and WT embryos were used, respectively, as positive and negative controls for the detection of the YFP expression. For the germ layer markers the following positive controls were used: fetal liver from at E13.5 embryo for the *Afp*, embryo at E9.5 for *Vimentin* and adult brain for *Tubb3*. PCR reactions were carried out as previously described^5^. Real-time PCR reactions for the pluripotency genes were carried out in triplicate with Lc Faststart Dna Masterplus Sybr (Roche) in a LightCycler II instrument (Roche), using 5 ng of cDNA and a 300 nM solution (final concentration) of specific forward and reverse primers. For the quantification, standard curves were prepared using 50 ng of cDNA from a pool of ESC serially diluted 1:5 for five standard points. Results are presented as the ratio of target gene mRNA content to housekeeping gene mRNA content expressed in arbitrary units. *B2m* was used as housekeeping gene. For primers specification see supplementary Table 2.

### Single cells deposition

Cells isolated from AF, at different embryonic stages, and AM were sorted for cKit expression as well as YFP^+^ ESC before and after IUT (isolated from AF and AM) were sorted for YFP expression using a FACS Aria I Sorter equipped with an automatic cell deposition unit (Becton Dickinson). 7AAD was added as a viability marker in the sorting procedure. Each cell was collected in single well of 96-well plates for molecular biology containing 5 μL of PBS-DEPC 0.1%, and stored at −80 °C.

### Single-cell multiplex PCR

We followed the protocol described in Franzin *et al.*^27^. Results are expressed as frequency of expression, that is the percentage of cells positive for each gene (*cKit*, *cMyc*, *Klf4*, *Nanog*, *Oct4*, *Sca1*, *Sox2*) on total analyzed cell number.

### Histology

Embryos obtained after IUT were washed in distilled water, dehydrated in ethanol gradient, left overnight in paraffin and sections were made using microtome.

Hematoxylin and eosin stain was performed with Hematoxylin/Eosin (HE) kit for rapid frozen section (Bio-Optica). Sections were observed by Olympus BX60 microscope (Olympus). Pictures were taken using Viewfinder Lite software.

### Immunofluorescence

Transverse sections of embryos or organs (7–10 μm thick) and AM tissue were fixed with 4% PFA and permeabilized with 0.5% triton-X 100 in PBS. The presence of YFP^+^ ESC was revealed using anti–GFP 594 antibody (1:150).

YFP^+^ ESC before and after IUT were fixed with 4% PFA and permeabilized using 0.1% NP-40. For staining have been used (1) the primary antibodies Oct4 (1:80), Sox2 (1:80) and Nanog (1:100) for cells; (2) cKit (1:80), αsmooth muscle actin (αSMA; 1:100), α fetoprotein (AFP; 1:200), βIII tubulin (Tubb3; 1:500), E-cadherin (1:100) for AM membrane; (3) anti–GFP 594 antibody (1:150) for organs and embryos, (4) Hoechst (1:1000, Life Technologies) for nuclei detention. Cells and tissue slides were observed under inverted immunofluorescence microscope (Leica DMI6000B, Leica Microsystems Srl). For antibodies specification see supplementary Table 3.

### Teratoma

For teratoma assay about 1 × 10^5^ or 1 × 10^6^ mouse YFP^+^ ESC (pre- and post-IUT; cells after IUT were collected from 100 embryos keeping distinguishing between those isolated from AF and AM) were injected into the muscle of the hindlimb of Rag2^−/−^γc^−/−^ mice straight after isolation from AF and AM digestion. After 6 weeks, mice were sacrificed and tumors collected for following analyses. Transverse sections (7–10 μm thick) of isopentan-frozen muscles were stained with hematoxylin and eosin to evaluate the tumor tissue composition.

For immunoperoxidase staining, teratoma sections were fixed using 4% paraformaldehyde (PFA) and permeabilized with 0.1% triton X-100. βIII tubulin (1:100), α fetoprotein (1:50) and αSMA (1:100) primary antibodies were diluted in 1% BSA in PBS and incubated for 1 hour at 37 °C (for βIII tubulin) or overnight at 4 °C (for others). After peroxidase blocking, secondary antibody HRP-conjugated was incubated for 45 minutes at 37 °C. After incubation with ImmPACT NovaRED (Vector Laboratories) for 5 minutes, cytoplasm was stained with Hematoxylin (Vector Laboratories) for 9 seconds.

For immunofluorescence analysis anti–GFP 594 antibody (1:150) and anti-Laminin antibody (1:100) were used. For antibodies specification see supplementary Table 3.

### Statistical analyses

Data were expressed as means ± s.e.m. Statistical significance was determined by using an equal-variance Student’s t-test and a p-value below 0.05 was considered to be statistically significant.

## Additional Information

**How to cite this article**: Bertin, E. *et al.* First steps to define murine amniotic fluid stem cell microenvironment. *Sci. Rep.* **6**, 37080; doi: 10.1038/srep37080 (2016).

**Publisher’s note:** Springer Nature remains neutral with regard to jurisdictional claims in published maps and institutional affiliations.

## Supplementary Material

Supplementary Information

## Figures and Tables

**Table 1:** Frequency of co-expression of pluripotency genes in mouse AFS cells.

	10.5	11.5	12.5	13.5	14.5
***ko***	0%	0%	10%	43%	0%
***ks***	0%	2%	13%	5%	0%
***km***	11%	18%	8%	12%	2%
***kom***	0%	0%	0%	9%	0%
***koms***	0%	0%	0%	0%	0%
***sm***	7%	2%	0%	2%	2%
***so***	0%	0%	0%	7%	0%
***om***	0%	0%	0%	33%	0%
***kos***	0%	0%	0%	5%	0%

***k*** = *Klf4*; ***m*** = *cMyc*; ***o*** = *Oct4*; ***s*** = *Sox2*.
