# Supplementary material for: First steps to define murine amniotic fluid stem cell microenvironment
Source: Sci Rep. 2016 Nov 15;6:37080. doi: 10.1038/srep37080 (PMC5109045; doi:10.1038/srep37080)
Supplement: Supplementary Information [file srep37080-s1.doc]

**First steps to define the murine amniotic fluid stem cell microenvironment**

Bertin E^1^, Piccoli M^1^, Franzin C^1^, Spiro G^1#^, Donà S^2^, Dedja A^3^, Schiavi F^4^, Taschin E^4^, Bonaldo P^2^, Braghetta P^2^, De Coppi P^5*^, Pozzobon M^1*^.

^1^ Stem Cells and Regenerative Medicine Lab, Fondazione Istituto di Ricerca Pediatrica Città della Speranza, Padova, Italy

^2^ Department of Molecular Medicine, University of Padova, Padova, Italy

^3^ Department of Cardiac, Thoracic and Vascular Sciences, University of Padova, Padova, Italy

^4^ Familial Cancer Clinic and Oncoendocrinology, Veneto Institute of Oncology, Padova, Italy

^5^ Stem Cells and Regenerative Medicine Section, Developmental Biology and Cancer Programme, UCL Institute of Child Health and Great Ormond Street Hospital, London, United Kingdom

^#^ Current affiliation: Department of Medicine - DIMED, University of Padova, Padova, Italy

^*^Corresponding author:

Paolo De Coppi

Stem Cells and Regenerative Medicine Section, Developmental Biology and Cancer Programme, UCL Institute of Child Health and Great Ormond Street Hospital, London, United Kingdom

e-mail: Paolo.DeCoppi@gosh.nhs.uk

Michela Pozzobon

Stem Cells and Regenerative Medicine Lab

Fondazione Istituto di Ricerca Pediatrica Città della Speranza, Padova, Italy

e-mail: m.pozzobon@irpcds.org

Tel: +39 0499640126

Fax: +39 0499640127

**
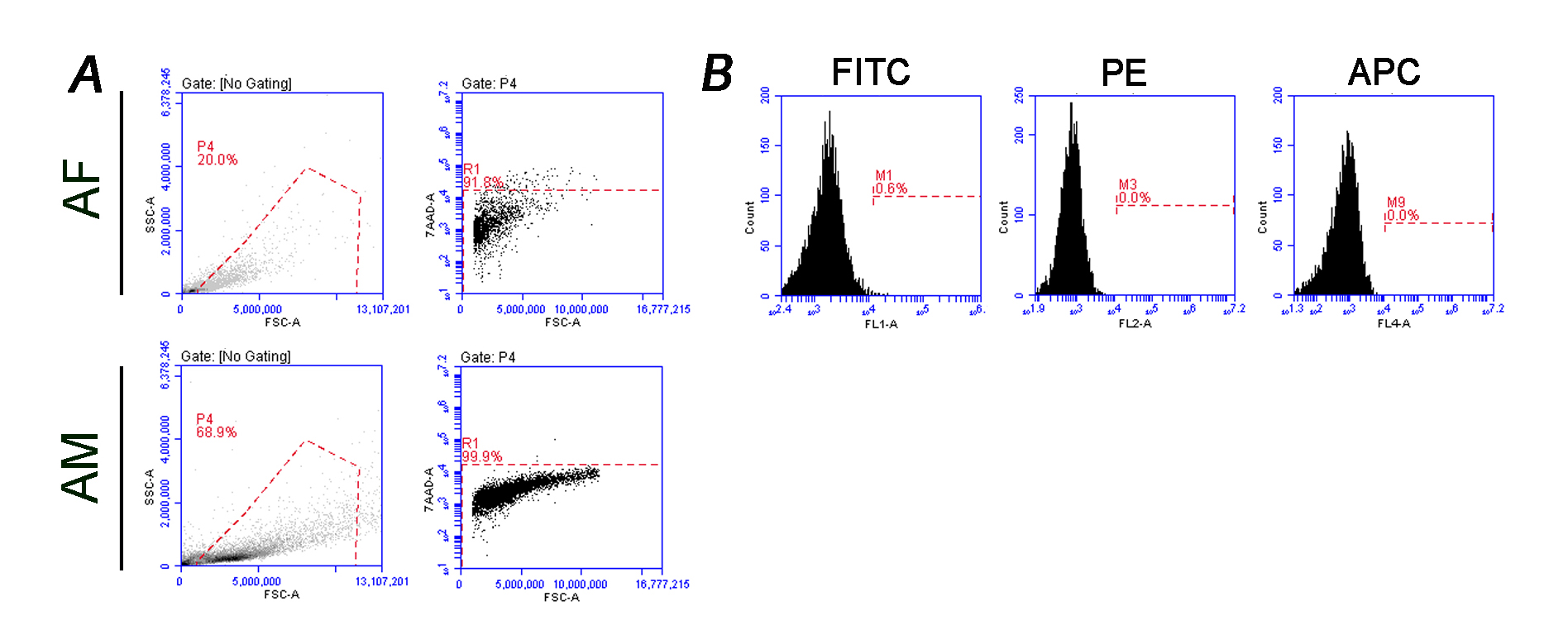
**

**Supplementary figure 1. Flow cytometry details.**

**(A):** After AF and AM cell collection, lineage negative cells (about 50.000 cells/tube) were incubated with selected antibodies conjugated alternatively with FITC, PE or APC fluorochromes. 7AAD was used as vitality exclusion markers. Briefly, in the morphological dot plot was gated the population of interest (P4), inside this population, only 7AAD negative cells were gated and considered for the specified marker expression analyses (R1). **(B):** Rat IgG 2a FITC-, rat IgG2a PE- and rat IgG2b APC**-**Isotype control were used to determine the degree of positivity.

**
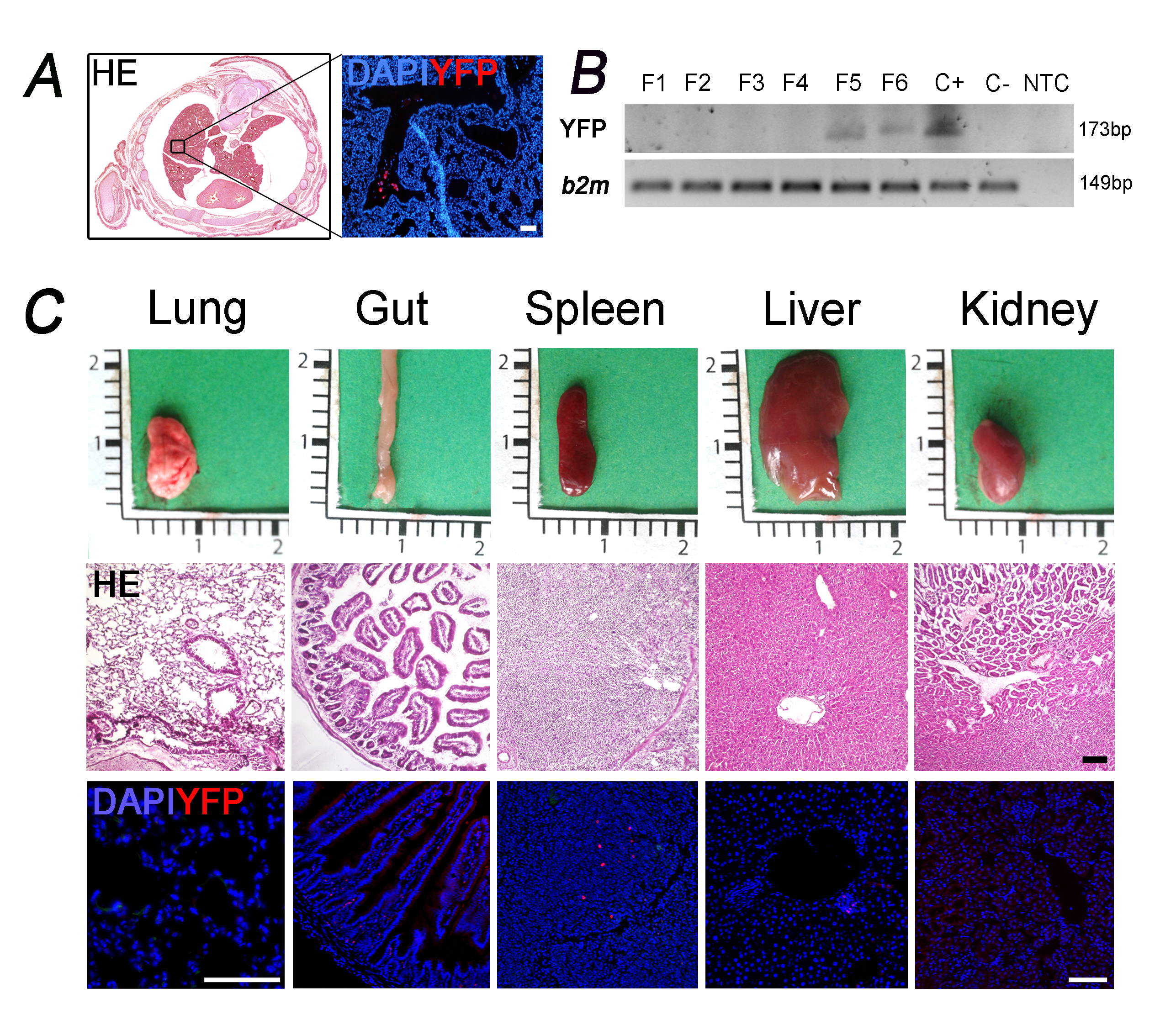
**

**Supplementary figure 2. Characterization of transplanted embryos.**

**(A):** Histological analysis of an upper part of an embryo obtained after IUT and immunostaining against YFP shows that YFP^+^ ESC did not integrate into tissues or organs (n=2 embryos). **(B):** Representative YFP PCR analysis of six embryos (samples from F1 to F6; total number of analyzed embryos = 13; 3 out of 13 were YFP^+^); C57Bl6/J-GFP^+^ embryo was used as positive control (C+) while wild type C57Bl6/J embryo as negative control (C-); NTC = not template control*. b2microglobulin* was used as housekeeping gene. **(C):** Morphological and immunostaining analyses revealed no YFP^+^ ESC integration in structures and tissues of transplanted embryos, evaluated 6 weeks after birth (n=3 mice). Scale bars = 100 μm.


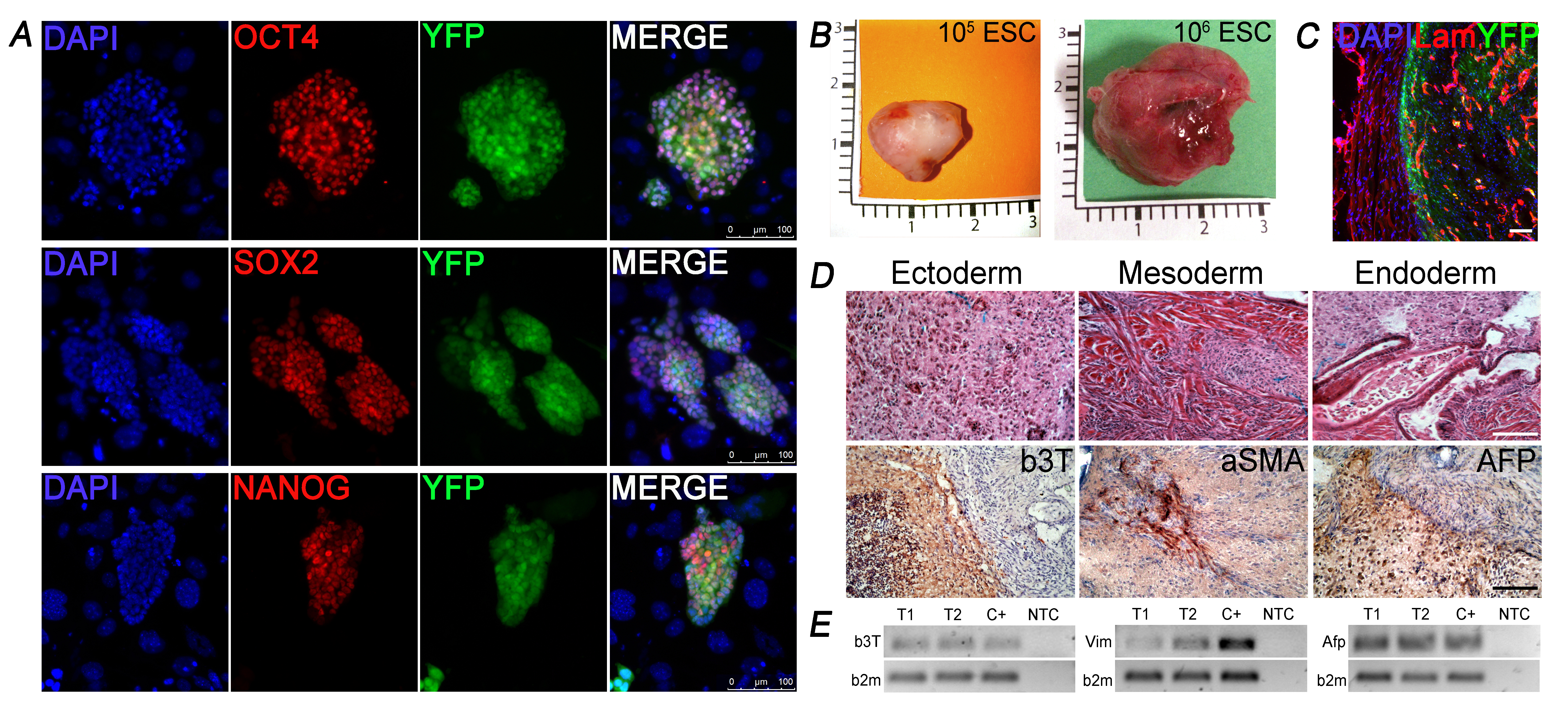


**Supplementary figure 3. Pluripotency of mouse YFP^+^ ESC before IUT.**

**(A):** Immunostaining for pluripotency markers of representative mouse YFP^+^ ESC colonies in culture, used for IUT experiments. **(B):** Size of the teratoma formed 6 weeks after injection of alternatively 10^5^ and 10^6^ YFP^+^ ESC into the hindlimb of Rag2^-/-^γc^-/-^ mouse. **(C):** Immunostaining for YFP and Laminin in teratoma confirmed that tumor masses originated from injected ESC. **(D):** Histology and immunostaining of teratoma validate the differentiation into all three germ layers (ectoderm, mesoderm and endoderm). **(E):** PCR analysis of two teratoma samples (T1 and T2) confirming the expression for *β3 tubulin*, *vimentin* and *αfetoprotein*. Scale bars = 100 μm.


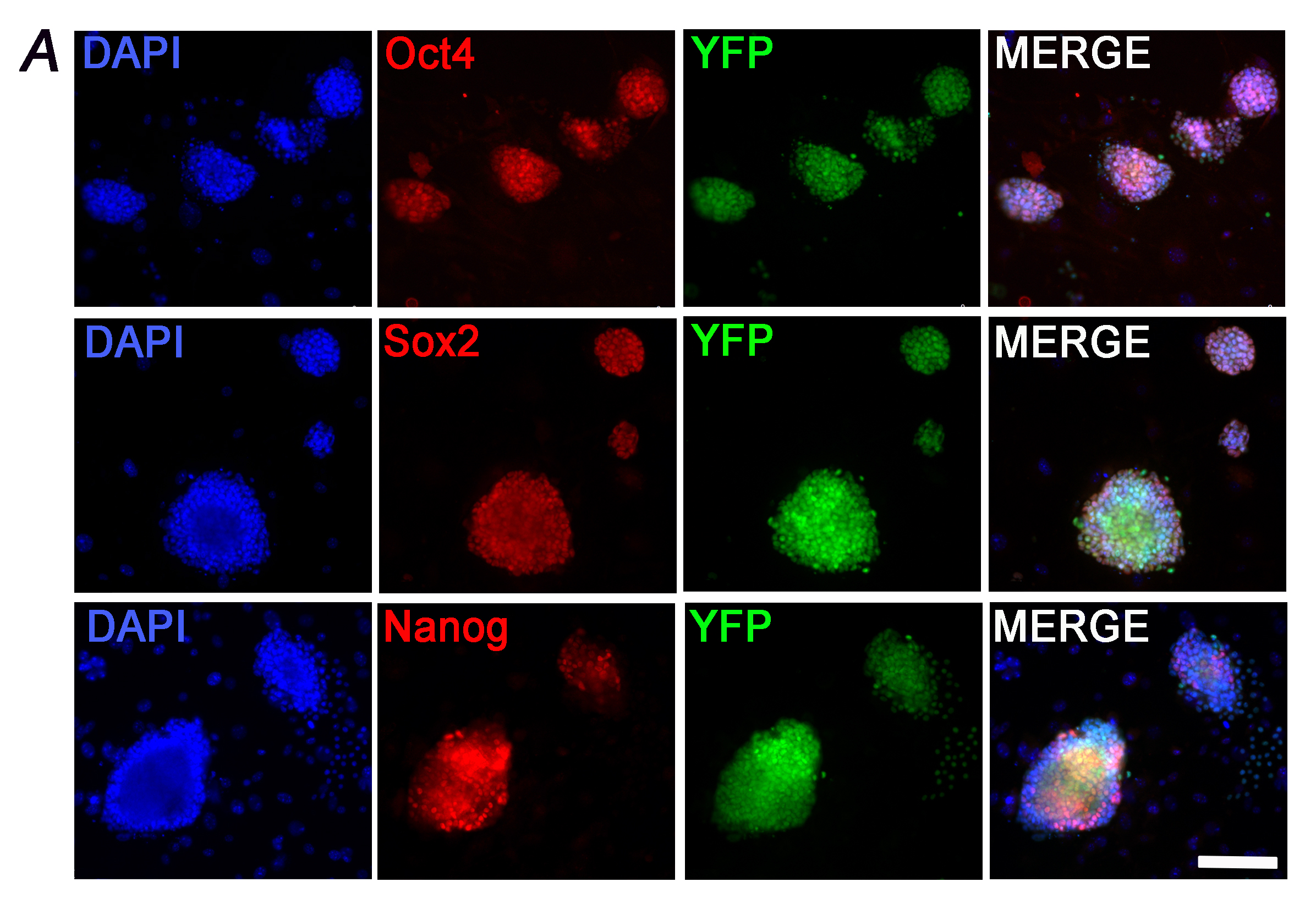


**Supplementary figure 4. *In vitro* expansion of YFP^+^ ESC on AM.**

**(A):** Immunostaining for pluripotency markers of YFP^+^ ESC expanded *in vitro* over E13.5 AM to simulate IUT condition. After 4 days, ESC displayed a phenotype very similar to standard culture appearance: they growth in colonies and expressed the pluripotency markers Oct4, Sox2 and Nanog.

**Supplementary table 1. IUT experiments and survival rate.**

|  | ***INJECTED***  ***EMBRYOS*** | ***SURVIVED***  ***EMBRYOS*** | ***OVERALL***  ***SURVIVAL*** | |
| --- | --- | --- | --- | --- |
| *IUT#1* | 8 | 8 | 100% | |
| *IUT#2* | 9 | 9 | 100% | |
| *IUT#3* | 7 | 7 | 100% | |
| *IUT#4* | 8 | 7 | 89% | |
| *IUT#5* | 10 | 9 | 90% | |
| *IUT#6* | 9 | 8 | 89% | |
| *IUT#7* | 10 | 8 | 80% | |
| *IUT#8* | 10 | 8 | 80% | |
| *IUT#9* | 9 | 2 | 22% | |
| *IUT#10* | 11 | 10 | 90% | |
| *IUT#11* | 10 | 7 | 70% | |
| *IUT#12* | 8 | 8 | 100% | |
| *IUT#13* | 8 | 8 | 100% | |
| *IUT#14* | 8 | 6 | 75% | |
| *IUT#15* | 11 | 5 | 45% | |
| *IUT#16* | 12 | 11 | 92% | |
| *IUT#17* | 10 | 8 | 80% | |
| *IUT#18* | 6 | 4 | 66% | |
| *IUT#19* | 12 | 8 | 67% | |
| *IUT#20* | 10 | 4 | 40% | |
| *IUT#21* | 7 | 7 | 100% | |
| *IUT#22* | 8 | 8 | 100% | |
| *IUT#23* | 10 | 10 | 100% | |
| *IUT#24* | 10 | 10 | 100% | |
|  | | ***SURVIVAL***  ***RATE*** | | **82%** |

**Supplementary table 2. RT-PCR primers used in this study.**

| **Gene** | **Forward** | **Reverse** |
| --- | --- | --- |
| *Oct4* | CCAACGAGAAGAGTATGAGGC | CAAAATGATGAGTGACAGACAGG |
| *Sox2* | TCTGTGGTCAAGTCCGAGGC | TTCTCCAGTTCGCAGTCCAG |
| *Nanog* | CCCTTCCCTCGCCATCACACTG | GGAAGGGCGAGGAGAGGCAGC |
| *Klf4* | GGCGAGAAACCTTACCACTGT | TACTGAACTCTCTCTCCTGGCA |
| *cMyc* | TGCCCGCGATCAGCTCTCCT | CGTGGCTGTCTGCGGGGTTT |
| *Tubb3* | TAGACCCCAGCGGCAACTAT | GTTCCAGGTTCCAAGTCCACC |
| *Vimentin* | CCAGAGACCCCAGCGCTCCT | GCCGGAGCCACCGAACATCC |
| *Afp* | GCTGTGGTGAGGGAATGGCCG | ACGAAGAGTTGCAGCAGTGGCT |
| *B2m* | GCTTCAGTCGTCAGCATGG | CAGTTCAGTATGTTCGGCTTCC |
| *GFP - YFP* | tgaaccgcatcgagctgaaggg | tccagcaggaccatgtgatcgc |

**Supplementary table 3. List of primary and secondary antibodies used.**

| **Primary Antibodies** | | | |
| --- | --- | --- | --- |
| **Antigen** | **Specie reactivity** | **Company** | **Cat.#** |
| Oct4 | Mouse Monoclonal | SantaCruz | sc-5279 |
| Sox2 | Goat Polyclonal | SantaCruz | sc-17320 |
| Nanog | Rabbit Polyclonal | Reprocell | RCAB0002P-F |
| cKit | Rabbit Polyclonal | SantaCruz | sc-5535 |
| AFP | Mouse Monoclonal | R&D | MAB1368 |
| Tubb3 | Mouse Monoclonal | Promega | G712A |
| αSMA | Mouse Monoclonal | Abcam | ab7817 |
| Laminin | Rabbit Polyclonal | Sigma | L9393 |
| E-Cadherin | Mouse Monoclonal | BD Biosciences | 610181 |
| Anti-GFP | Rabbit Polyclonal | Life Technologies | A21312 |
| **Secondary Antibodies** | | | |
| **Name** | **Label or Dye** | **Company** | **Cat.#** |
| Anti-Rabbit | Alexa Fluor 594 | Life Technologies | A21442 |
| Anti-Goat | Alexa Fluor 594 | Life Technologies | A21468 |
| Anti-Mouse | Alexa Fluor 594 | Life Technologies | A11005 |
| Anti-Mouse | HRP | SantaCruz | sc-2005 |
